# The Impact of Autologous Platelet Concentrates on the Periapical Tissues and Root Development of Replanted Teeth: A Systematic Review

**DOI:** 10.3390/ma15082776

**Published:** 2022-04-10

**Authors:** Zohaib Khurshid, Faris Yahya I. Asiri, Shariq Najeeb, Jithendra Ratnayake

**Affiliations:** 1 Department of Prosthodontics and Dental Materials, School of Dentistry, King Faisal University, Al-Ahsa 31982, Saudi Arabia; 2 Department of Preventive Dentistry, College of Dentistry, King Faisal University, Al-Ahsa 31982, Saudi Arabia; fasiri@kfu.edu.sa; 3 Schulich School of Medicine and Dentistry, University of Western Ontario, London, ON N6A 3K7, Canada; snajeeb3@uwo.ca; 4 Faculty of Dentistry, Sir John Walsh Research Institute, University of Otago, P.O. Box 56, Dunedin 9054, New Zealand; jithendra.ratnayake@otago.ac.nz

**Keywords:** tooth replantation, autologous platelet, platelet-rich fibrin, platelet-rich plasma, tooth avulsion

## Abstract

Introduction: In many cases, the replanted teeth may undergo resorption or ankyloses. Recent studies show that autologous platelet concentrates (APCs) may improve the outcomes of tooth replantation. The aim of this systematic review was to summarize and critically appraise the currently available literature on the use of APCs before tooth replantation. Methodology: An electronic search was conducted on the following research databases: PubMed/MEDLINE, ISI Web of Science, EMBASE and Scopus. The following medical subject heading (MeSH) keywords used were: ((tooth replantation) OR (replanted tooth) OR (teeth replantation) OR (replanted teeth)) AND ((autologous platelet concentrate) OR (platelet-rich plasma) OR (platelet-rich fibrin) OR (autologous platelet)). The studies’ data was extracted, and the research’ quality was rated using the CARE and ARRIVE protocols. Results: Ten case reports and three animal studies, one cell study and one study, which included both animal and in vitro experiments, were included in this review. In majority of the studies, APCs improved the outcomes of tooth replantation. However, there were various sources of bias in the most of the research, which may have influenced the results. Conclusions: Although majority of the studies indicate that APCs may improve outcomes of tooth replantation, majority of the studies contained numerous sources of bias. Additionally, the sample size of the included subjects is inadequate to predict the clinical efficacy of APCs in management of replanted teeth. Large-scale, multi-center and long-term studies are required to ascertain the efficacy of APCs in improve the outcomes of tooth replantation.

## 1. Introduction

Avulsion of a tooth occurs when it is completely dislodged from its socket as a result of trauma [1]. Replanting teeth that have been left out extra-orally for more than 60 min is very unlikely to survive, hence immediate replantation is the best therapy for tooth avulsion. Delaying replantation for more than fifteen minutes reduces the success probability of tooth replantation [2]. Additionally, in many cases, immediate replantation of the avulsed tooth is not possible, and tooth may be either kept in inappropriate storage media or left out to dry, leading to necrosis of the pulp and damage to the attached periodontal tissue on the root [1]. If quick replantation is not possible, the avulsed tooth should be stored in a suitable medium, such as saliva, milk, or Hank’s Balanced Salt Solution (HBSS) [3,4]. However, due to the lack of awareness or unavailability of such media, many patients or their guardians bring the avulsed tooth in clinical improperly handled. Therefore, delayed replantation of teeth can lead to many complications such as resorption of the root and periapical periodontal tissues, ankylosis of the tooth and necrosis of the pulp [2]. According to studies, the rate of inflammatory root resorption following tooth replantation is as high as 23%, while the rate of replacement root resorption is around 51% [2]. To improve the outcomes of delayed tooth replantation, several treatments may be carried out. Pulpectomy involves the removal of the potentially necrotic pulp that may cause internal resorption [5]. Additionally, after pulpectomy calcium hydroxide (CH) paste or mineral trioxide aggregate may be placed in the canal for several days to prevent resorption and promote the formation of a calcific barrier in immature teeth with an open apex [6]. Furthermore, surface treatment with anti-resorptive agents such as sodium fluoride may decrease the likelihood of root or bone resorption post replantation [7]. Other drugs, such as bisphosphonates [8] and growth factors, such as fibroblast growth factor-2 (FGF-2) and enamel matrix derivative [9], have recently been studied for their potential anti-resorptive and regenerative effects, but no large-scale studies documenting their clinical efficacy have been published to date.

Autologous platelet concentrates (APCs) are produced by centrifuging the patient’s own blood and injecting or topically injecting the isolated plasma, which is rich in grown factors, regenerative cells and leukocytes, into the wound or defect [10]. In dentistry, two generations of APCs have been studied. Platelet-rich plasma (PRP) are first generation APCs that are produce by double-spin centrifuging of the blood [11]. PRPs contain a high concentration of plarelets and growth factors that have been used to promote wound healing and periodontal regeneration. On the other hand, the second-generation platelet-rich fibrin (PRF) is produced by single-spin centrifuging and has the fibrin matrix network intact [11]. Efficacy of platelet concentrates in promoting wound healing and tissue regeneration is at the center of a recent academic debate [12]. Systematic reviews indicate that APCs promote root development and apical closure in immature or young permanent teeth [13,14,15]. Similarly, recently published case reports and animal studies indicate that APCs may improve the outcomes of tooth replantation [16,17]. However, no systematic review summarizing the outcomes and appraising quality of the literature focusing on the effect of ACs on the outcomes of tooth replantation has been published. Therefore, the objectives of this systematic review are: (1) to summarize the outcomes of APC application on replanted teeth reported in literature and (2) to appraise the quality of the literature focusing on using APCs to improve the outcomes of tooth replantation.

## 2. Materials and Methods

### 2.1. Focused Question and Registration

Using the Participants, Interventions, Controls, Outcomes (PICO) principle defined in the Preferred Reporting Items for Systematic Reviews and Meta-analysis (PRISMA) statement [18], the following focused question was constructed: ‘Does the application of autologous platelet concentrates improve the outcomes of tooth replantation?’. The protocol of the systematic review was registered on PROSPERO (Reg no. CRD42021292877).

### 2.2. Eligibility Criteria

Three investigators (ZK, FA and SN) agreed on pre-defined eligibility criteria before commencing the literature search. All types of clinical studies (randomized clinical trials, observational studies, cohort studies and case studies/series), in vitro (cell) studies, and animal studies, published during or after the year 1990 and reporting the periodontal and tooth- or root-related outcomes after using APC were included. Reviews, commentaries, letters to the editor and studies not in English were excluded.

### 2.3. Literature Search

**Figure 1:**
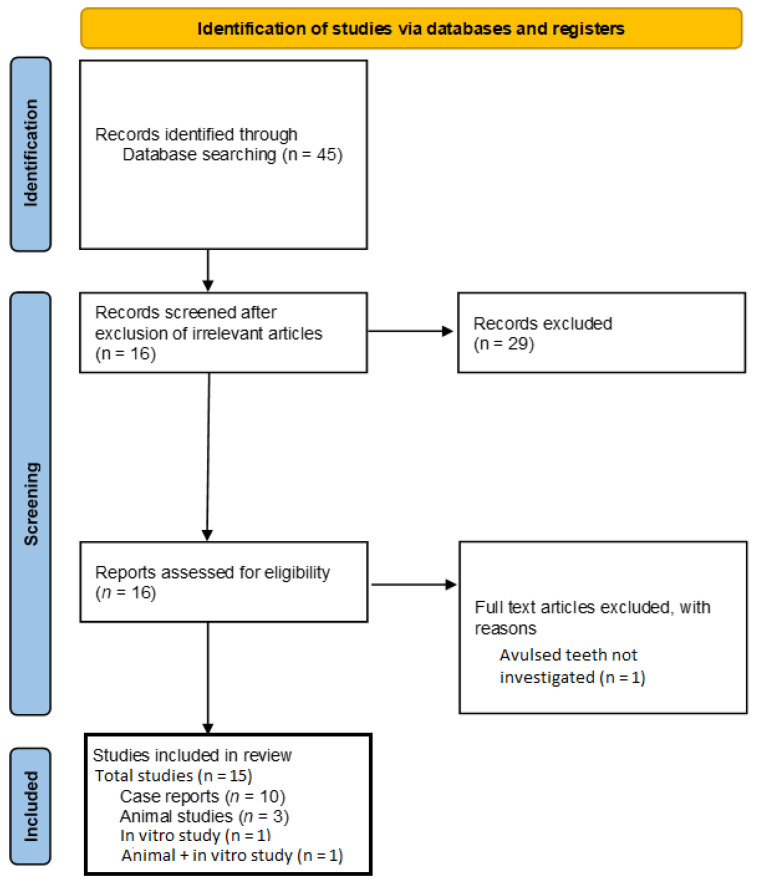
A PRISMA flow diagram for the search methodology employed for this systematic review.

PubMED/MEDLINE, ISI Web of Science, Embase, and Scopus were used to conduct an electronic search. The following medical subject heading (MeSH) keywords used were: ((tooth replantation) OR (replanted tooth) OR (teeth replantation) OR (replanted teeth)) AND ((autologous platelet concentrate) OR (platelet-rich plasma) OR (platelet-rich fibrin) OR (autologous platelet)). The abstracts and titles of the studies obtained after the primary search were read to exclude irrelevant studies. Full texts of the potentially eligible studies were downloaded and were read comprehensively. Additionally, the reference lists of these studies were read to find any additional articles relevant to this review. Two investigators (ZK and FA) carried out the whole literature search, and any disputes were resolved through conversation. The search methodology is peresented in Figure 1.

### 2.4. Data Extraction

The two investigators extracted information from the studies into tables according to the PICO principle. Two different tables were created because there were two different types of studies included. From the case reports, following data was extracted: age and gender of the patient treated, number and type of tooth replanted, status of apical closure, reason for replantation, type and site of APC, periodontal parameters reported, total follow up and the outcomes. The data from animal studies belonging to the following elements was extracted: species and number of animals included, type and number of teeth replanted, treatment groups, site of APC application, extra-oral time experienced by the replanted teeth, periodontal variables assessed, duration of the study and the overall outcomes. Any disagreements were solved by discussion and the tabulated data was validated by a third investigator (FYA). The various categories of data extracted are provided in Table 1 and Table 2.

### 2.5. Quality Assessment

The quality of the case reports were assessed using the Preferred Reporting Items for Case reports in Endodontics (PRICE) guidelines [19]. Briefly, the quality assessment of the following elements was carried out: the title, keywords, abstract, introduction, patient information, clinical findings, timeline, diagnostic assessment, intervention details, follow-up, outcomes, discussion, patient perspective and informed content. The qualitative assessment of the animal studies and in vitro experiments was carried out using the Preferred Reporting Items for study Designs in Endodontology (PRIDE) [20]. For the quality assessment of animal studies, the following study attributes were reviewed: study design, sample size, inclusion and exclusion criteria, randomization, blinding, outcome measures, statistics, details of the experimental animals and the quality of the results. Each study was given an overall quality score of ‘low’, ‘medium’ or ‘high’ depending on the quality scale attributes fulfilled. The attributes assessed for the quality assessment are presented in additional supplementary files. The quality assessment was carried independently by the two reviewers (ZK and SN). Any disagreements were solved by discussion.

## 3. Results

### 3.1. Results of the Literature Search

The initial literature search resulted in 45 articles. After the elimination of 29 articles on the basis of titles and abstracts, 16 full-text documents were downloaded. One study was purely in vitro [21] and one study included both in vitro and animal experiments [22]. Of these 16 articles, one article was excluded because it did not involve avulsed teeth [23]. Therefore, 15 studies were included in this review for qualitative and quantitative assessment [16,17,21,22,24,25,26,27,28,29,30,31,32,33,34]. Among these included studies, 10 studies were case reports [16,24,25,26,27,28,29,30,31,32], 3 were animal studies [17,33,34]. No studies were found in the reference lists of the included studies. The PRISMA flow diagram for the literature search is presented in Figure 1.

### 3.2. General Characteristics of the Case Reports

In all the 10 case reports, one tooth was replanted per patient [16,24,25,26,27,28,29,30,31,32]. Therefore, 10 teeth (5 maxillary central incisors [26,28,29,30,31], 2 mandibular incisors [24,25], 2 maxillary secondary premolars [16,32] and 1 mandibular first molar [30] were replanted in as many patients [16,24,25,26,27,28,29,30,31,32]. 8 patients were male [16,24,25,26,27,28,29,32] and 2 patients were female [30,31] and age of the patients ranged from 11 to 45 years [16,24,25,26,27,28,29,30,31,32]. In 7 studies, apical closure been completed [24,25,26,29,30,31,32] and in 2 studies the apex was open at the time of APC application [16,28]. In 1 study, the status of apical closure was not stated [27]. In 2 studies, teeth were intentionally replanted due to periodontal bone loss and mobility [24,25,29] and in 3 studies, teeth were replanted because they had avulsed [26,28,31]. One case report described the replantation of a tooth that had been accidentally extracted and had undergone pulpal necrosis [16]. In one study, a tooth that had previously undergone extrusive luxation and had become mobile was replanted [27]. In one case report, replantation following root resection due to a separated instrument had been described [30] and in one case report, intentional replantation of a tooth in which vertical root fractured had occurred was described [32]. PRP was used in 5 studies [16,24,25,27,28] and PRF was used in 5 studies [26,29,30,31,32]. In 4 studies, PRF was applied in the socket [29,30,31,32] and in one study intracanal PRF was used [26]. In two studies, PRP was applied in the socket [24,25] and in two studies it was used in the root canal [16,28]. In one study, PRP was mixed with nano-hydroxyapatite (nano-HA) and placed in the socket [27]. Four studies just reported radiographic assessment [27,28,29,32] and two studies assessed radiographic outcomes and mobility [26,31]. In one study, pulp status in addition to radiographic outcomes was assessed [16]. In one study, periodontal probing and mobility assessment was caried out [25]. In one study, periodontal probing, radiography and mobility assessment was carried out [24]. Seven case reports reported follow up in months which ranged from 5.5 to 18 months [16,24,25,26,27,28,29]. 3 case reports reported a follow up duration of 1–2 years [30,31,32]. The general characteristics of the case reports are presented in Table 1.

### 3.3. General Characteristics of Animal Studies

All animal studies replanted teeth in dogs [17,33,34]. Two studies used adult mongrel dogs [17,33] and in one study, neither the developmental stage nor the breed of the dogs was stated [32]. The number of animals ranged from 2 to 6 [17,33,34]. In one study, 64 in incisors and premolars were replanted [33] and in another study 16 incisors and premolars were replanted [17]. In two studies, APC was applied in the socket [17,33] and in one study, it was used on the root surface [34]. In one study, 36 incisor and premolar roots were replanted [34]. Extra-oral times before tooth replantation were 30 and 60 min in two studies [17,33]. In one study the extra-oral time was 5 min but the PDL and cementum was removed in two experimental groups [34]. One study evaluated effects of the following on tooth replantation: PPP (platelet-poor plasma), calcium chloride–activated PRP (PRP/Ca), calcium chloride- and thrombin-activated PRP (PRP/Thr/Ca), and bone marrow mononuclear cells and PRP/Ca (BMMCs/PRP/Ca) [33]. In one study, the efficacy of PRP was tested on replantation of roots in which PDL and cementum was removed and the outcomes were compared to effect of saline on roots with intact PDL and cementum and roots with PDL and cementum removed [34]. In one study, the effect of PRF was compared to no treatment [17]. The number of teeth replanted were stated as 8 and 12 in two studies [17,34] and in one study, the number was stated [33]. All studies assessed periodontal regeneration, root resorption and ankylosis by means of histological assessment [17,33,34] while in one study, immunohistochemistry was used to analyze osteoprotegerin (OPG), collagen III synthesis and laminin synthesis, and tartrate-resistant acid phosphatase (TRAP) was used to assess osteoclast activity [33]. The general characteristics of the animal studies are detailed in Table 2.

### 3.4. General Characteristics of In Vitro Experiments

In the first in vitro study, human PDL stem cells (hPDLSCs) were cultured with PRF and the expressions of bone sialoprotein (BSP), alkaline phosphatase (ALP), osteocalcin (OCN) and collagen-1 (coll-1) were measured using immunohistochemistry [22]. In the second study in vitro study, viable PDL cells attached on 30 extracted teeth were treated with PRF and PPP (plasma-poor plasma) after 40 min of drying time [21]. The comparison groups were teeth treated with PPP treatment only after 40 min of drying, no drying time and no treatment and those teeth that undergone an hour of drying and not treated. The general characeristics of in vitro experiments are listed in Table 2.

### 3.5. Outcomes of the Included Studies

In nine case reports, radiographic outcomes were reported [16,24,25,26,27,28,29,30,32]. In these studies, there was improvement in periapical bone regeneration after APC application as evident by reduction or resolution of periapical radiolucencies [16,24,25,26,27,28,29,30,32]. In one study, improvement in alveolar bone levels was detected on follow up [24,25]. In one study, a reduction of mobility from Class III to Class II was observed [24]. Finally, in one study, application of PRP with MTA in a previously necrosed pulp resulted in sensitivity on follow up, indicating return of pulp vitality [16]. In one study, no clear outcomes were reported at follow-up [31]. The detailed outcomes of the case reports are presented in Table 1. In all animal studies, APC-based treatment improved the outcomes of tooth replantation [17,33,34]. In one study, only PRP/Thr/Ca reduced root resorption and PPP, PRP/Ca and BMMCs/PRP/Ca application resulted root resorption [33]. In one animal study, PRF was combined with dog hPDLSCs to induce higher bone and PDL regeneration [22]. In one study, PRP reduced ankylosis and induced formation of PDL- and cementum-like [34]. In both the in vitro studies, PRF induced a higher proliferation PDL cells [21,22]. In one study, significantly less inflammatory root resorption was observed in the teeth replanted after PRF therapy [17]. The detailed outcomes of the animal reports and the in vitro experiments are presented in Table 2.

### 3.6. Overall Quality of Included Studies

Overall, four case reports were awarded a grade of ‘high’ [24,26,28,30], three studies received a grade of ‘medium’ [25,29,32] and two case reports were deemed to be having a low quality [27,31]. On the other hand, two animal studies received a grade of ‘high’ and one animal study was graded as ‘medium’ [17,33,34]. One in vitro study received a grade of ‘high’ [22] and another received a grade of ‘medium’ [21]. The detailed results of the quality assessment of the individual characteristics of the case reports and animal studies are provided in Table 3 and Table 4 respectively. The quality assessment results of in vitro experiments are provided in Table 5.

## 4. Discussion

APCs have been utilized to treat pattern baldness [35] and enhance wound healing because to their regenerative capacity [36]. Their regenerative potential has been mainly attributed to the high concentration of platelets. Platelets emit many regenerative growth factors, including platelet-derived growth factor (PDGF), transforming growth factor (TGF), and insulin-like growth factor-1 and -2 (IGF-1 and -2), which have been shown to enhance periodontal tissue regeneration [37,38,39]. Hence, it is not surprising that APCs have been studied for their potential in improving the outcomes of periodontal regeneration [40]. Similarly, three systematic reviews have been published that have focused on appraising and summarizing the literature on using APCs in regenerative endodontics [13,14,15]. Studies included in these reviews suggest that APCs may promote root formation and apical closure in immature teeth that have been treated endodontically [13,14,15]. However, in each of these reviews, only one study (by Priya et al. [28]) describing replantation of a tooth had been included. To the best of the authors’ knowledge, this is the first systematic review that has focused on studies that have looked at the outcomes of APCs after replantation.

Replantation of teeth may be carried out as a first line treatment of traumatically avulsed teeth [41] or to improve the prognosis of periodontally compromised or hopeless teeth [42]. In this systematic review, autologous platelet concentrates have been used to improve the outcomes of replantation in both the scenarios [16,24,25,26,27,28,29,30,32]. Using APCs to improve the outcomes of replantation is not new. A 1986 study by Nasjleti et al. observed effect of PRP on the cellular proliferation on and around teeth replanted 5 min after extraction in monkeys [43]. Although this study was not included the qualitative analysis in our systematic review because it was published before 1990, it is still noteworthy to mention that the proliferative effects of autologous platelet have been documented in literature published more than three decades ago. In vitro studies have attempted the study the effect of APCs on avulsed teeth. Hiremath et al. studied the effects of PRF on the PDL cells attached on extracted teeth that been tried for an hour. They observed that a combined use of PPP and PRF stimulated the proliferation of PDL cells [21]. In vitro experiments by Zhou et al. have attempted to use autologous platelet in combination with stem cells to regenerate the attached periodontal tissue on avulsed teeth [22]. In the same study the authors observed that PRF induced a higher expression of ALP, OCN, BSP and coll-1—all of which are biomarkers of bone and PDL regeneration [22]. However, the in vivo or clinical potential of using both APCs and stem cells are yet to be seen. Nevertheless, in all the case reports reviewed in this systematic review, autologous platelet usage resulted in favourable outcomes and no adverse effects were reported [16,24,25,26,27,28,29,30,31,32].

Perhaps, the most significant observation in the case reports is the amount of bone regeneration post-replantation after APC application. Tözüm et al. (2005) reported a decrease of a Class III mobility to Class I [24] and an improvement in bone levels of as much as 6 mm was reported by Demit et al. [25] after applying PRF in the socket. This bone regenerative potential of PRF has a significant clinical potential in improving the outcomes of teeth replanted because of severe periodontal bone loss or mobility. This is most likely because PRF has been observed to stimulate pro-osteoblastic factor RUNX and a reduction in the mineralization inhibitor MGT in vitro [44]. Furthermore, clinical studies suggest that a combined use of PRF and xenografts may improve outcomes of bone augmentation [45]. Similarly, one case report in this review described the use of PRP in combination of a xenograft and a collagen membrane [29]. Therefore, PRP, in combination with other regenerative materials, holds potential in regenerating bone around periodontally compromised teeth that have been intentionally replanted. PRF is a second-generation APC and it is produced by a single spin during centrifugation [21] which is in contrast to the double-spin centrifugation needed to produce PRP [10]. The advantage of PRF is that the fibrin network is still intact and can release growth factor for a period of 7 to 14 days which is significantly better than the growth factor release duration of 14 h from PRP [12]. Due to its superior space-maintenance and mechanical integrity compared to PRP, PRF has an added advantage of functioning as a guided tissue regeneration (GTR) membrane. Nevertheless, one study has found no significant difference between the efficacy of PRP and PRF in periodontal regeneration [46]. Therefore, more studies are required before the superiority of PRF over PRP can be ascertained.

Replantation of teeth that have been left out extra-orally for extended periods of time present a particular challenge due to several factors. Firstly, dehydration of the periodontal tissues attached to the root leads to the necrosis of regenerative cells that play a vital role in periodontal healing [47]. Furthermore, necrosis of the pulp tissue adversely affects the outcomes of tooth [1]. Also, bacterial contamination of the pulp and/or the root also leads to failure of the replantation process [48]. Therefore, root resorption and ankylosis are common complications following delayed replantation [49]. Loss of periodontal support caused by periapical bone loss following root replantation can also lead to failure of tooth replantation [50]. Although a number of pre-replantation intracanal and root surface treatments have been advocated to improve the outcomes, prevalence of root resorption and ankylosis following delayed replantation remains high [49]. Three case reports analyzed APCs for their regenerative properties when used as an intracanal medicament after delayed replantation [26,28,31]. In another case report, although PRP was placed in the canal, the pulp vitality was not evaluated or reported [28]. Therefore, in that study, it is unknown what effect if any, did APC have on pulp tissues. The authors of another case report a positive pulp test at 24-month follow up which indicates that PRF may stimulate regeneration of the radicular pulp tissue to an extent [26]. Torabinejad and Turman (2011) report that using PRP not only results in healing of periapical tissues but also results in regeneration of vital tissues in a root canal that had previously contained necrosed pulp [16]. However, in this case the follow up period was only 5.5 month and it is unknown if subsequent follow up was carried out to reaffirm this return of pulp vitality. None of the three animal studies included in this review attempted to assess the pulp-related outcomes of APC [17,33,34]. Therefore, to date, there is insufficient evidence from clinical and pre-clinical studies to ascertain the efficacy of APCs in regenerating pulp tissues.

There have been no large-scale or long-term clinical investigations evaluating the efficacy of APCs on the results of replanted teeth to yet. Quality assessment of the case reports revealed numerous additional limitations. Firstly, in each case report, there was only one patient treated [16,24,25,26,27,28,29,30,31,32]. Hence, only 10 replanted teeth, after being treated with APC, have been documented in the literature to date. This small sample is insufficient to conclude the effectiveness of APCs in improving the outcomes of tooth replantation. Furthermore, majority of the case reports [25,27,28,29,30,31,32] did not report if any of the patients treated had any other comorbidities or congenital disorders. Therefore, it is unknown if similar successful results would be observed if the teeth were replanted (after being treated with APCs) in patients who are otherwise systemically unhealthy. Only four studies declared any or no conflicts of interests or their sources of funding behind the studies [16,26,27,30] which could mean that the remaining six studies may have a high likelihood of funding or experimenters’ bias. Similarly, only one animal study out of the three included in this review employed any blinding to reduce any sources of observer’s bias [17]. Additionally, none of the animal studies included in this review stated whether there were any animals or teeth lost during the experiments. Although animal models are widely used to study the effect of interventions in periodontology and endodontics, due to the difference in the microflora and dietary patterns between animals and humans, it is difficult to predict to certainty that the results from in vivo studies will translate to clinical practice [51].

As with all pre-replantation regimens, the main factor governing the success of replantation is the extra-oral drying time the tooth may undergo following avulsion [5]. An attractive aspect of APCs as a regenerative material that, because it is dervided from the host’s own blood, it has an extremely low probability of rejection. To date, no reports have documented any adverse reactions caused by APCs themselves. Nevertheless, one case report has recorded an allergic reaction following PRP therapy which was most likely due to calcium citrate, an anticoagulant that is added to APCs [52]. The data regarding the safety of APC when used periodontally or intracanal is scarce. Moreover, in other fields or surgery and medicine, studies have found inconclusive evidence regarding the safety of APCs. Another clinical aspect that the future studies should focus on long-term safety of the APC procedures in endodontics and tooth replantation. Clinically, APCs may be applied either on the root, the socket or both but no study has compared the efficacy of these routes. In majority of the case reports included in this review, APCs were applied in the socket (extraradicular) and only two studies documented the intracanal use of APCs in open apices before replantation which warrants further research focusing on the usage of APCs in the apexogenesis of immature teeth [16,28]. Therefore, it is evident that there is a lack of standardization of APCs application on replanted teeth. Furthermore, the relatively short follow-up time of the studies makes the long term efficacy of APCs debatable [16,17,21,22,24,25,26,27,28,29,30,31,32,33,34]. Therefore, further studies should evaluate the intracanal efficacy of APC in improving the outcomes of tooth replantation. In particular, long-term randomized controlled trials are essential to ascertain the clinical efficacy of APCs as pre-replantation treatment.

In additional to the limitations of the included studies, this systematic review has some limitations as well. Due to the heterogenous nature of the animal studies and the lack of control groups in the case reports, it was not possible to conduct a meta-analysis and only a qualitative assessment of the studies was possible. Therefore, the overall mean effect of PRP on pulpal or periodontal regeneration compared to conventional intracanal medicaments such as calcium hydroxide or surface treatments is unknown. Future clinical studies should compare the efficacy of different types of APCs such as PRP and PRP with each other. Furthermore, large scale trials are necessary to determine if APCs are superior to existing pre-replantation treatment of teeth. Similarly, future animal studies should focus on the differences between the regenerative abilities of APCs and other commonly used intracanal medicaments such as calcium hydroxide and MTA, and surface treatments such as sodium fluoride.

## 5. Conclusions

Within the limitations of this systematic review, it may be concluded that autologous platelet concentrates may improves the outcomes of tooth replantation, in particular root formation, apical closure and pulpal regeneration. APCs may prove to be important substitutes or adjuncts to existing pre-replantation endodontic, root surface or socket treatments. However, large-scale clinical studies and better-designed animal experiments are required before APCs may see wider use in dental traumatology.

## Figures and Tables

**Table 1:** General characteristics and outcomes of the case reports describing the use of autologous platelet therapy to improve the outcomes of tooth replantation.

Study	Patient Details (Gender, Age)	Tooth (n)	Apex (Open/Close)	Reason for Replantation	Type of Autologous Platelet and Location	Other Treatment	Clinical Parameters Assessed	Follow Up	Outcome (s)
Tözüm et al., 2005	Male, 42 years	Mand central incisor	Closed	Periodontitis	PRP, socket	RCT, periodontal therapy	Periodontal probing, radiography, mobility	18 months	Mobility decreased to Grade I from Grade III after 12 months of PRP therapy. 3–3.5 mm of new alveolar bone detected.
Demir et al., 2007	Male, 45 years	Mand central incisor	Closed	Periodontitis	PRP, socket	RCT, periodontal therapy, GTR with PTFE and BG graft	Periodontal probing, radiography	12 months	Bone fill detected radiographically. Pocket depths reduced from 8-7-10 (distal-median-mesial) on the buccal side and 7-7.9 mm on the lingual side to 3-3-4 and 3-3.4 mm respectively.
Johns et al., 2011	Male, 15 years	Max central incisor	Closed	Avulsion (8 h storage in milk)	PRF, intracanal	RCT, root resection	Radiography, mobility	24 months	PT results positive. Normal mobility and thick radiopacity surrounding area of root resection.
Torabinejad & Turman 2011	Male, 11 years	Max second premolar	Open	Accidental extraction (immediately replanted); pulpal necrosis.	PRP + MTA, intracanal	Antibiotic paste, RCT	Pulp status, Radiography	5.5 months	Continued root development and apical closure; complete resolution of PA radiolucency; return of pulpal sensitivity.
Patel et al., 2013	Male, 16 years	Max central incisor	Not stated	Mobile (extrusive luxation)	PRP (mixed with nano HA), socket	RCT, periodontal therapy	Radiography	6 months	Reduction in radiolucency.
Priya et al., 2015	Male, 11 years	Max central incisor	Open	Avulsion (>8 h extra-oral dry time)	PRP, intracanal	Antibiotic paste, splinting, RCT	Radiography	12 months	Complete resolution of PA radiolucency. Normal mobility.
Ryana et al., 2016	Male, 23 years	Max central incisor	Closed	Periodontitis (post trauma)	PRF, socket	RCT, xenograft, Type I collagen membrane	Radiography	12 months	87% new bone detected radiographically.
Deshpande et al., 2019	Female, 23 years	Mand first molar	Closed	Separated instrument in root canal	PRF, socket	RCT, root resection	Periodontal examination, radiography	2 years	No periodontal pathologies detected clinically or radiographically.
Suresh 2019	Female, 21 years	Max central incisor	Closed	Avulsion (>4 days extra-oral time)	PRF, socket	RCT, splinting	Radiography, mobility	1 year	No outcomes reported.
Yang et al., 2021	Male, 20 years	Max second premolar	Closed	Vertical root fracture	PRF, socket	RCT, endodontic surgery	Radiography	2 years	Reduction in PA radiolucency

**Table 2:** General characteristics and outcomes of the animal studies and in vitro studies included in this review.

Study	Type of Study	Animals (*n*)/In Vitro Model	Teeth Analyzed	Groups (*n*)	Site of APC	Extra-Oral Time/Tooth Storage	Periodontal Variables Assessed	Duration of Study	Outcomes
Assuncao et al., 2011	Animal	Adult dogs (*n* = 4)	Premolars (*n* = 64)	1: No treatment 2: PPP 3: PRP/Ca 4: PRP/Thr/Ca 5: BMMCs/PRP/Ca (*n* not stated)	Socket	30 min	Histologic, histomorphometric, and immunohistochemical analysis (OP, TRAP, Type III Col, laminin)	120 days	Only PRP/Thr/Ca group did not exhibit root resorption. All other groups displayed replacement or inflammatory resorption.
Zhao et al., 2013	In vitro/Animal	In vitro: hPDLCSs Animal: Dogs (*n* = 6)	Incisors (*n* = 36)	In vitro: hPDLSC + PRF No treatment Animal: 1: Dog PDLSCs + PRF 2: DogThe format of n is not uniform, there are both regular and italic, please unify the full textPDLCSs 3: Dog PRF 4: Dog PDLSCs	Socket	2 h	In vitro: ALP, BSP, Col-1, OCN Animal: Histology—bone and PDL regeneration	In vitro: 7 days Animal: 8 weeks	PRF/PDLSC grafts induced higher PDL proflieration and regeneration of bone and PDL tissue.
Hiremath et al., 2014	In vitro	PDL cells on extracted human teeth	Unspecified (*n* = 30)	1: PRF + PPP (40 min dry) 2: PPP (40 min dry) 3: 0 min, no treatment 4: 1 h, no treatment		0 min, 40 min, 1 h	PDL cell count		PRF + PPP stimulated higher PDL cell proliferation,
Yang et al., 2018	Animal	Mongrel dogs (*n* = 6; breed and gender not stated)	36 roots from premolars and incisors (*n* = 36)	1: Saline (PDL and cementum intact) (*n* = 12) 2: Saline (PDL and cementum removed) (*n* = 12) 3: PRP (PDL and cementum removed) (*n* = 12)	Intracanal	5 min	Histological, histomorphometric analysis	8 weeks	PRP reduced ankylosis and promoted PDL- and cementum-like tissue formation significantly more than Group 2.
Behnaz et al., 2021	Animal	Adult mongrel dogs (*n* = 2)	Incisors and premolars (*n* = 16)	1: No treatment (*n* = 8) 2: PRF (n = 8)	Socket	60 min	Histological examination	8 weeks	Less inflammatory resorption in PRF group. No significant difference in periodontal tissues between groups.

**Table 3:** Results of the quality assessment of the included case reports. The details of each study item assessed is provided in the additional supplementary tables.

Section/Topic	Item Number	Tözüm et al., 2005	Demir et al., 2007	Johns et al., 2011	Torabinejad & Turman 2011	Patel et al., 2013	Priya et al., 2015	Ryana et al., 2016	Deshpande et al., 2019	Suresh 2019	Yang et al., 2021
Title	1a	No	Yes	No	Yes	No	Yes	No	No	No	Yes
	1b	Yes	Yes	Yes	Yes	Not clear	Yes	Yes	Yes	Yes	Yes
Keywords	2a	No	Yes	Yes	Yes	Yes	Yes	Yes	Yes	Yes	Yes
Abstract	3a	Yes	Yes	Yes	Yes	No	Yes	Yes	Yes	No	Yes
	3b	Yes	No	Yes	Yes	No	Yes	Yes	Yes	No	Yes
	3c	Yes	No	Yes	Yes	No	Yes	Yes	Yes	No	Yes
	3d	Yes	No	Yes	Yes	No	Yes	Yes	Yes	No	Yes
	3e	Yes	No	No	Yes	No	Yes	Yes	Yes	No	Yes
Introduction	4a	Yes	Yes	Yes	Yes	No	Yes	Yes	Yes	Yes	Yes
Informed consent	5a	Yes	Yes	No	Yes	No	Yes	Yes	Yes	Yes	No
Case report information	6a	Yes	Yes	Yes	Yes	Yes	Yes	Yes	Yes	Yes	Yes
	6b	Yes	Yes	Yes	Yes	Yes	Yes	Yes	Yes	Yes	Yes
	6c	No	No	No	No	No	No	No	No	No	No
	6d	Yes	No	Yes	Yes	Yes	Yes	Yes	Yes	Yes	Yes
	6e	Yes	No	Yes	No	No	Yes	No	Yes	No	No
	6f	Yes	Yes	Yes	Yes	Yes	Yes	Yes	Yes	Yes	Yes
	6g	N/A	N/A	N/A	N/A	N/A	N/A	N/A	N/A	N/A	N/A
	6h	No	No	No	No	No	No	No	No	No	No
	6i	Yes	No	Yes	Yes	No	No	No	No	No	No
	6j	No	No	Yes	Yes	No	Yes	No	Yes	No	No
	6k	Yes	Yes	Yes	Yes	Yes	Yes	No	Yes	Yes	Yes
	6l	No	No	No	No	No	No	No	No	No	No
	6m	Yes	Yes	Yes	Yes	Yes	Yes	No	No	No	No
	6n	No	No	No	No	No	No	No	No	No	No
	6o	No	No	No	Yes	No	No	No	No	No	No
	6p	Yes	Yes	Yes	Yes	Yes	Yes	Yes	Yes	Yes	No
	6q	No	Yes	Yes	No	Yes	No	Yes	No	No	Yes
	6r	No	No	No	No	No	No	No	No	No	No
	6s	No	No	No	No	No	No	No	No	No	No
Discussion	7a	Yes	Yes	Yes	Yes	Yes	Yes	Yes	Yes	No	Yes
	7b	No	No	No	No	No	Yes	No	No	No	No
	7c	No	Yes	No	Yes	No	Yes	No	No	No	No
	7d	Yes	Yes	No	Yes	No	Yes	Yes	Yes	No	Yes
Patient perspective	8a	Yes	Yes	No	Yes	Yes	No	No	No	No	No
Conclusion	9a	Yes	Yes	No	Yes	Yes	Yes	Yes	Yes	No	Yes
	9b	Yes	Not clear	No	Yes	Yes	Yes	Yes	Yes	No	Yes
Funding details	10a	No	No	No	No	Yes	No	No	Yes	No	No
Conflict of interest	11a	No	No	Yes	Yes	Yes	Yes	No	Yes	No	Yes
Quality of clinical images and radiographs	12a	No	No	No	No	No	No	No	No	No	No
	12b	Yes	Yes	Yes	Yes	Yes	Yes	Yes	Yes	Yes	Yes
	12c	No	No	No	No	No	No	No	No	No	No
	12d	No	No	No	No	No	No	No	No	No	No
	12e	Yes	Yes	Yes	Yes	Yes	Yes	Yes	Yes	Yes	Yes
	12f	Yes	Yes	Yes	Yes	Yes	Yes	Yes	Yes	Yes	Yes
	12g	Yes	Yes	Yes	Yes	Yes	Yes	Yes	Yes	Yes	Yes
	12h	Yes	Yes	Yes	Yes	Yes	Yes	Yes	Yes	Yes	Yes
	12i	Yes	Yes	Yes	Yes	Yes	Yes	Yes	Yes	Yes	Yes
Overall quality		High	Medium	High	High	Low	High	Medium	High	Low	Medium

**Table 4:** Results of the quality assessment of the included anmal studies. The details of each study item assessed is provided in the additional supplementary file.

Section/Topic	Item Number	Assuncao et al., 2011	Zhao et al., 2013	Yang et al., 2018	Behnaz et al., 2021
Title	1a	No	No	Yes	No
	1b	Yes	Yes	Yes	Yes
Keywords	2a	No	Yes	No	No
Abstract	3a	Yes	Yes	Yes	Yes
	3b	Yes	Yes	Yes	Yes
	3c	Yes	Yes	Yes	Yes
	3d	Yes	Yes	Yes	Yes
	3e	Yes	Yes	Yes	Yes
	3f	Yes	Yes	Yes	Yes
Introduction	4a	Yes	Yes	Yes	Yes
	4b	No	No	No	No
	4c	Yes	Yes	Yes	Yes
	4d	Yes	Yes	Yes	Yes
Materials and Methods	5a	Yes	Yes	Yes	Yes
	5b	No	No	No	Yes
	5c	No	No	No	Yes
	5d	No	No	No	No
	5e	No	No	Yes	Yes
	5f	No	No	Yes	Yes
	5g	Yes	Yes	Yes	Yes
	5h	Yes	Yes	Yes	Yes
	5i	No	No	No	Yes
	5j	Yes	Yes	Yes	Yes
	5k	Yes	Yes	Yes	Yes
	5l	No	No	No	Yes
Results	6a	Yes	No	Yes	Yes
	6b	Yes	Yes	Yes	Yes
	6c	No	No	No	No
	6d	Yes	Yes	Yes	Yes
Discussion	7a	Yes	Yes	Yes	Yes
	7b	No	No	No	No
	7c	Yes	Yes	Yes	Yes
	7d	Yes	Yes	Yes	Yes
Conclusion(s)	8a	Yes	Yes	Yes	Yes
	8b	Yes	Yes	Yes	Yes
Funding and support	9a	Yes	Yes	Yes	Yes
Conflicts of interest	10a	Yes	Yes	Yes	Yes
Quality of images	11a	Yes	Yes	Yes	Yes
	11b	No	No	No	No
	11c	No	No	No	No
	11d	Yes	Yes	Yes	Yes
	11e	Yes	Yes	Yes	Yes
	11f	Yes	Yes	Yes	Yes
	11g	Yes	Yes	Yes	Yes
	11h	Yes	Yes	Yes	Yes
Overall quality		Medium	High	High	High

**Table 5:** Results of the quality assessment of the in vitro experiments reported by the included studies.

Topic	Item Number	Zhou et al., 2013	Hiremath et al., 2014
Title	1a	No	Yes
	1b	Yes	Yes
Keywords	2a	Yes	Yes
Abstract	3a	Yes	Yes
	3b	Yes	Yes
	3c	Yes	Yes
	3d	Yes	Yes
	3e	Yes	Yes
Introduction	4a	Yes	Yes
	4b	Yes	Yes
Materials and Methods	5a	Yes	No
	5b	No	No
	5c	Yes	Yes
	5d	Yes	No
	5e	Yes	Yes
	5f	No	No
	5g	No	No
	5h	Yes	Yes
	5i	No	No
	5j	Yes	No
Results	6a	Yes	Yes
	6b	No	No
	6c	Yes	Yes
Discussion	7a	Yes	Yes
	7b	Yes	Yes
	7c	No	No
	7d	No	No
	7e	Yes	Yes
Conclusion(s)	8a	Yes	No
	8b	Yes	Yes
Funding and support	9a	Yes	Yes
Conflicts of interest	10a	Yes	Yes
Quality of images	11a	Yes	No
	11b	Yes	No
	11c	Yes	No
	11d	Yes	No
	11e	Yes	Yes
	11f	Yes	No
	11g	Yes	No
	11h	Yes	Yes
Overall quality		High	Medium

## Data Availability

Not applicable.

## Supplementary Materials

The following supporting information can be downloaded at: https://www.mdpi.com/article/10.3390/ma15082776/s1; Table S1: The items assessed in the included case reports using the PRICE guidelines; Table S2: The items assessed in the animal studies included in this review using the PRIASE guidelines.
