# Supplementary material for: The Impact of Autologous Platelet Concentrates on the Periapical Tissues and Root Development of Replanted Teeth: A Systematic Review
Source: Materials (Basel). 2022 Apr 10;15(8):2776. doi: 10.3390/ma15082776 (PMC9031947; doi:10.3390/ma15082776)
Supplement: Supplementary file 1 [file materials-15-02776-s001.zip › materials-1653239-supplementary.pdf]

Table S1. The items assessed in the included case reports using the PRICE guidelines.

Section/Topic	Item Number	Checklist Item
**Title**	1a	The words "case report(s)" must be included in the title
	1b	The area of interest (e.g. anatomy, disease, treatment) must be included briefly in the title
**Keywords**	2a	At least two relevant keywords, preferably MeSH terms, related to the content of the case report must be included
**Abstract**	3a	The Introduction must contain information on how the report is novel and contributes to the literature, clinical practice and/or fills a gap(s) in knowledge
	3b	The Body must describe the main clinical findings, including symptoms and signs, if present
	3c	The Body must describe the main radiographic/histological/ laboratory/diagnostic findings
	3d	The Body must describe the main outcomes of treatment, if active treatment has been provided
	3e	The Conclusion(s) must contain the main "take-away" lesson(s), sometimes referred to as key learning point(s)
**Introduction**	4a	A background summary of the case(s) with relevant information must be provided
**Informed consent**	5a	A clear statement that informed, valid consent was obtained from the patient(s) must be provided
**Case report information**	6a	The age of the patient(s) must be provided
	6b	The gender of the patient(s) must be provided
	6c	The ethnicity of the patient(s) must be provided, if relevant
	6d	The main concern, chief complaint or symptoms of the patient(s), if any, must be provided
	6e	The medical history of the patient(s) must be provided, if relevant
	6f	The dental history of the patient(s) must be provided, if relevant
	6g	The family history of the patient if associated with the primary complaint must be provided, if relevant
	6h	The psychosocial history of the patient if associated with the primary complaint must be provided, if relevant

	6i	Genetic information, including details of relevant comorbidities and past interventions and their outcomes must be provided when possible, if relevant
	6j	Extra-oral findings must be provided, if relevant
	6k	General intra-oral findings must be provided when relevant, e.g. carious lesions, restorations, periodontal condition, soft tissues etc.
	6l	Important/relevant dates and times (in the text, or a table or figure) must be provided in chronological order
	6m	The diagnostic methods and the results for the specific tooth/teeth (e.g. pulp sensibility test, tenderness, mobility, periodontal probing depths, laboratory investigations, imaging techniques, or other special tests) must be provided
	6n	The diagnostic challenges, if any, must be provided
	6o	The diagnostic reasoning including other possible diagnoses that were considered must be provided
	6p	The active treatment (s) or intervention(s) performed, if any, must be provided
	6q	Any modifications to the proposed treatment(s) or intervention(s), if necessary, must be provided
	6r	The assessment method(s) used to determine the clinician-assessed and patient-assessed treatment outcomes and their results must be provided
	6s	Adverse and unanticipated events or consequences, if any, must be provided
**Discussion**	7a	The specific treatment(s) and intervention(s) (if any) must be discussed with reference to the relevant literature
	7b	The strengths of the case report and its importance must be discussed with reference to the relevant literature
	7c	The limitations of the case report must be discussed
	7d	The rationale for the conclusion(s) must be discussed
**Patient perspective**	8a	Feedback from the patient on the treatment and the care they received should be provided, if relevant
**Conclusion**	9a	Explicit conclusion(s), i.e. the main “take-away” lessons must be provided
	9b	Implications for clinical practice or future research must be provided

**Funding details**	10a	Sources of funding and other support (such as supply of instruments, equipment) as well as the role of funders must be acknowledged and described
**Conflict of interest**	11a	An explicit statement on conflicts of interest must be provided
**Quality of images**	12a	Details of the equipment, software and settings used to acquire the image(s) must be described in the text or legend
	12b	The reason why the image(s) was acquired and the rationale for its inclusion in the manuscript must be provided in the text
	12c	The circumstances (conditions) under which the image(s) were viewed and evaluated by the authors must be provided in the text
	12d	The resolution and any magnification of the image(s) or any modifications/enhancements (e.g. adjustments for brightness, colour balance, or magnification, image smoothing, staining etc.) that were carried out must be described in the text or legend
	12e	Patient(s) identifiers (names, patient numbers) must be removed to ensure they are anonymised
	12f	An interpretation of the findings (meaning and implications) from the image (s) must be provided in the text
	12g	The legend associated with each image must describe clearly what the subject is and what specific feature(s) it illustrates. Legends associated with images of patients must describe the age, gender and ethnicity of the person, if relevant
	12h	Markers/labels must be used to identify the key information in the image(s) and be defined in the legend or as a footnote
	12i	The legend of each image must include an explanation whether it is pre-treatment, intra-treatment or post-treatment and, if relevant, how images over time were standardised

Table S2. The items assessed in the animal studies included in this review using the PRIASE guidelines.

Section/ Topic	Item Number	Checklist Items
Title	1a	The specific animal species and its health or disease status (sometimes called “animal model”) must be provided.
	1b	The specific test, field, subject and treatment of interest within the animal model must be provided.
Keywords	2a	Keywords such as “animal model” or “*in vivo* model” and the specific area(s) of interest must be provided.
Abstract	3a	The Introduction of the Abstract must explain the significance of the study.
	3b	The unambiguous aim(s) and objective(s) of the study must be provided.
	3c	The most important details of the animal and the experimental model must be provided.
	3d	Key details of the methodology must be provided.
	3e	The most relevant and important results must be presented succinctly including differences among the means, medians or modes of the dependent variables (treatment outcome and test results) and any significant P-values.
	3f	Succinct conclusions supported by the results must be provided.
Introduction	4a	The relevant background information must be provided using terminologies consistent with professional standards and previous publications.
	4b	The appropriateness of the selected animal model to address the aims and objectives of the study must be explained.
	4c	A justification of the reasons why the investigation was necessary using an animal model must be provided.
	4d	The unambiguous aim(s) and objectives(s) of the animal study must be provided.
Materials and Methods	5a	The reference number of the approval granted by the ethics board, such as an Institutional Review Board or Institutional Animal Care committee, must be provided along with a reference to the applicable institutional and/or national regulations that were enforced. Any identifying details about the authors institution should not be disclosed during the blind peer review.

	5b	The sample size must be justified by citing prior similar studies and/or be estimated by using statistical power calculations to ensure an adequate sample size is used to detect any significant differences and answer the research questions. This is to avoid making any type I and type II errors.
	5c	Details of how animal pain and disability was monitored and how animal suffering was prevented during all aspects of experimentation must be provided.
	5d	The job titles and qualifications of the animal caretakers must be provided.
	5e	Specific details of the animals must be provided, including their species, strain, immune system, breeding programme, age, weight, health status, and any special characteristics.
	5f	The experimental design must include details of the numbers of animals, numbers of experimental units (e.g. teeth), and timelines (e.g. 5, 30 and 60 days) used.
	5g	The primary outcome data measures or categories as well as any other secondary outcome data measures or categories that will be assessed must be provided.
	5h	Details must be provided on (1) steps in the interventions and treatments and (2) instruments, medicaments or device allocation.
	5i	Details regarding post-disease and post-operative care of the animals must be provided.
	5j	Details on the statistical analysis, statistical tests, type of software used, and steps taken to control, interpret success or failure, and to validate the accuracy of the data must be provided.
	5k	Randomization.
	5l	Blinding.
Results	6a	Average baseline characteristics of the animals (e.g. age, weight, gender, microbiological status) at the beginning of the experiment must be provided.
	6b	The results for each group of primary and secondary outcomes should describe the means, median or mode; as well as differences and their statistical significance.
	6c	All adverse events during the animal experimentation and the method of euthanasia must be reported.

	6d	Any changes made to the experimental protocols to prevent the occurrence of animal adverse health events, analgesic or other medication overdoses or underdoses, or unexpected deaths must be provided.
Discussion	7a	A discussion on how the methods and results are relevant to the study aims, and how the results support or dispute prevailing theories advocated in prior publications must be provided.
	7b	An objective presentation of the strengths and limitations of the animal model, study design, methods, materials, instruments, drugs and devices, and outcomes must be provided, including any biology/functional variability between the animal model and humans.
	7c	The potential influence of the results on future research plans must be discussed.
	7d	If appropriate, the impact the findings have on human health, treatments or healthcare must be explained.
Conclusion(s)	8a	A rational basis for the conclusion(s) must be provided, that is, they must be directly supported by the results of the study.
	8b	Explicit conclusion(s) from the study, including appropriate follow-up research ideas, must be provided.
Funding and support	9a	All funding, donations, assistance and support provided for the study must be reported.
Conflicts of interest	10a	An explicit statement on conflicts of interest must be provided.
Quality of images	11a	Details of the equipment (model, supplier, city, country), software (version, supplier city, country) and settings used to acquire image(s) must be described in the Methods and/or figure legend.
	11b	The reason why the image(s) was acquired and rationale for its inclusion in the manuscript must be provided in the text.
	11c	The circumstances (conditions) under which the image(s) was viewed and evaluated must be provided in the text.
	11d	The resolution, magnification and any important manipulation(s) on any image (e.g. brightness, image smoothing, staining etc.) must be described in the text or legend.

	11e	An interpretation of the findings (meaning and implications) from the image (s) must be provided in the text.
	11f	The legend associated with each image must clearly describe the subject matter specific feature(s) illustrated. Images of animals must describe their age and test duration, and other relevant features such as important anatomical landmarks and relevant features.
	11g	Arrow markers and relevant labels must be provided in image(s), if relevant, in order to identify key information.
	11h	The legend of each image must include an explanation whether it refers to pre-treatment, intra-treatment, post-treatment or post-sacrifice, and if relevant, how images were standardised over time.
